# Establishment of anti-DKK3 peptide for the cancer control in head and neck squamous cell carcinoma (HNSCC)

**DOI:** 10.1186/s12935-022-02783-9

**Published:** 2022-11-15

**Authors:** Naoki Katase, Shin-ichiro Nishimatsu, Akira Yamauchi, Shinji Okano, Shuichi Fujita

**Affiliations:** 1 Department of Oral Pathology, Graduate School of Biomedical Sciences, Nagasaki University, 1-7-1 Sakamoto, Nagasaki, Nagasaki 852-8588 Japan; 2 Department of Natural Sciences, Kawasaki Medical School, Kurashiki, Okayama 701-0192 Japan; 3 Department of Biochemistry, Kawasaki Medical School, Kurashiki, Okayama 701-0192 Japan; 4 Department of Pathology, Nagasaki University Hospital, 1-7-1 Sakamoto, Nagasaki, Nagasaki 852-8501 Japan; 5 Department of Pathology, Graduate School of Biomedical Sciences, Nagasaki University, 1-7-1 Sakamoto, Nagasaki, Nagasaki 852-8501 Japan

**Keywords:** DKK3, Head and neck squamous cell carcinoma, Inhibitory peptide

## Abstract

**Background:**

Head and neck squamous cell carcinoma (HNSCC) is the most common malignant tumor of the head and neck. We identified cancer-specific genes in HNSCC and focused on DKK3 expression. DKK3 gene codes two isoforms of proteins (secreted and non-secreted) with two distinct cysteine rich domains (CRDs). It is reported that DKK3 functions as a negative regulator of oncogenic Wnt signaling and, is therefore, considered to be a tumor suppressor gene. However, our series of studies have demonstrated that DKK3 expression is specifically high in HNSCC tissues and cells, and that DKK3 might determine the malignant potentials of HNSCC cells via the activation of Akt. Further analyses strongly suggested that both secreted DKK3 and non-secreted DKK3 could activate Akt signaling in discrete ways, and consequently exert tumor promoting effects. We hypothesized that DKK3 might be a specific druggable target, and it is necessary to establish a DKK3 inhibitor that can inhibit both secreted and non-secreted isoforms of DKK3.

**Methods:**

Using inverse polymerase chain reaction, we generated mutant expression plasmids that express DKK3 without CRD1, CRD2, or both CRD1 and CRD2 (DKK3ΔC1, DKK3ΔC2, and DKK3ΔC1ΔC2, respectively). These plasmids were then transfected into HNSCC-derived cells to determine the domain responsible for DKK3-mediated Akt activation. We designed antisense peptides using the MIMETEC program, targeting DKK3-specific amino acid sequences within CRD1 and CRD2. The structural models for peptides and DKK3 were generated using Raptor X, and then a docking simulation was performed using CluPro2. Afterward, the best set of the peptides was applied into HNSCC-derived cells, and the effects on Akt phosphorylation, cellular proliferation, invasion, and migration were assessed. We also investigated the therapeutic effects of the peptides in the xenograft models.

**Results:**

Transfection of mutant expression plasmids and subsequent functional analyses revealed that it is necessary to delete both CRD1 and CRD2 to inhibit Akt activation and inhibition of proliferation, migration, and invasion. The inhibitory peptides for CRD1 and CRD2 of DKK3 significantly reduced the phosphorylation of Akt, and consequently suppressed cellular proliferation, migration, invasion and in vivo tumor growth at very low doses.

**Conclusions:**

This inhibitory peptide represents a promising new therapeutic strategy for HNSCC treatment.

**Supplementary Information:**

The online version contains supplementary material available at 10.1186/s12935-022-02783-9.

## Background

Head and neck squamous cell carcinoma (HNSCC) is the most common malignancy of the head and neck region, including the oral cavity, larynx, pharynx, nasal cavity, and paranasal sinuses. Its incidence is increasing both in developing countries and developed countries. HNSCC is the sixth most common cancer worldwide, with 890,000 new cases and 450,000 deaths reported in 2018 [1]. HNSCC is believed to arise due to cumulative abnormalities in the cancer-associated genes, which may be caused by alcohol consumption, smoking, betel quid chewing or viral infections. Recent studies have demonstrated that there are two distinct types of HNSCC, according to the presence or absence of human papilloma virus (HPV) infection: HPV-positive HNSCC and HPV-negative HNSCC. The former is commonly observed in the oropharynx, tonsils and base of the tongue, typically occuring in younger patients who are often non-smokers [2], and is characterized by a solid sheet-like, trabecular proliferation of cancer cells without keratinization; while the latter commonly occurs in the oral cavity and larynx associated with smoking and alcohol consumption, in which the cancer nest shows structured keratinization. There are differences between the two, not only in the histological characteristics and frequent sites, but also in the genetic background. It has been reported that E6 and E7 viral proteins from HPV-16 cause the functional loss of TP53 and Rb tumor suppressors in HPV-positive HNSCC, and that HPV-positive HNSCC often lacks mutation in TP53, which would explain the far more favorable outcome of HPV-positive HNSCC [3, 4]. Moreover, recent studies suggested the existence of a subgroup of genetically distinct HPV-negative HNSCC with favorable prognoses [4, 5]. HNSCC includes highly heterogeneous cases with different oncogenic initiation events and distinct genetic profiles.

Recent studies using genomics and big data analyses have elucidated the genetic background of HNSCC. In 2015, the Cancer Genome Atlas (TCGA) consortium published a comprehensive genetic characterization of HNSCC [5]. To date, some gene mutations have been shown to be important in the tumorigenesis, invasion, and metastasis of HNSCC. For instance, mutations in TP53, loss of CDKN2A, and amplification of CCND1 have been reported in HPV-negative HNSCC, and PI3KCA amplification is commonly observed in HNSCC regardless of HPV infection status [2, 4, 5]. Nevertheless, novel druggable oncogenes have not been identified to date, and these overwhelming molecular data should be interpretated biologically.

In this context, we have pursued the cancer-specific molecules that could be possible druggable targets, and have focused on the DKK3 gene [6]. DKK3 belongs to the Dickkopf WNT signaling pathway inhibitor family, which consists of DKK1, DKK2, DKK3, and DKK4. The DKK family members encode secretory proteins with two distinct cysteine rich domains (CRDs), which function as endogenous Wnt/β-catenin signaling inhibitors [7]. DKK1, DKK2, and DKK4 inhibit Wnt signaling by binding to the receptor Kremen1/2, and inducing the internalization of LRP5/6 by endocytosis. Although DKK3 cannot bind to Kremen1/2, the cytoplasmic isoform of DKK3 (DKK3b) shuts off the nuclear translocation of β-catenin and exert Wnt inhibitory function [7, 8]. In addition to its Wnt inhibitory function, DKK3 is also characterized as a tumor suppressor because of its reduced expression in many types of malignancies [8, 9]. However, our series of studies has demonstrated that the DKK3 function in HNSCC is completely different and indicates complex oncogenic functions. We have shown that in many cases of HNSCC, DKK3 protein expression is high and is associated with poorer prognosis [10–13], and that transient knockdown of DKK3 in HNSCC-derived cells resulted in significantly reduced cellular migration and invasion [12]. Moreover, DKK3 over-expression resulted in significantly elevated tumor cell proliferation, migration, invasion and in vivo tumor growth via increased Akt phosphorylation [14], whereas stable knockdown of DKK3 showed completely opposing effects in HNSCC cells [15]. Our data strongly suggest that DKK3 might exert oncogenic functions specifically in HNSCC, and DKK3 could be a target for molecular targeted therapy. Our results also imply that it is important to inhibit protein–protein interactions between DKK3 and its effector proteins simultaneously in both secreted and cytoplasmic isoforms of DKK3.

Therefore, we established an inhibitory peptide that specifically binds DKK3 and inhibits its oncogenic function. Based on the comparative-genome analyses, we hypothesized that the two CRDs of DKK3 might be functionally important for protein interaction. In this study, we generated deletion mutants of DKK3 expression plasmids that enables the expression of DKK3 lacking CRD1 (DKK3ΔC1), CRD2 (DKK3ΔC2), or both CRD1 and CRD2 (DKK3ΔC1ΔC2), and evaluated its effects on cellular proliferation, migration, and invasion. Moreover, we designed specific complementary peptides for CRDs and tested whether these peptides could suppress the oncogenic function of the DKK3 protein.

## Methods

### Cell lines

The human tongue cancer-derived cell line, HSC-3 was used in this study and was purchased from RIKEN Bioresource Center (Tsukuba, Japan). The cells were maintained in Dulbecco’s modified Eagle’s medium (DMEM; FUJIFILM Wako Pure Chemical Corporation, Osaka, Japan), supplemented with 10% fetal bovine serum (FBS; Cosmo Bio Co., Ltd., Tokyo, Japan).

### Inverse polymerase chain reaction (PCR) for the generation of DKK3 deletion mutant plasmids, and transfection

**Fig. 1:**
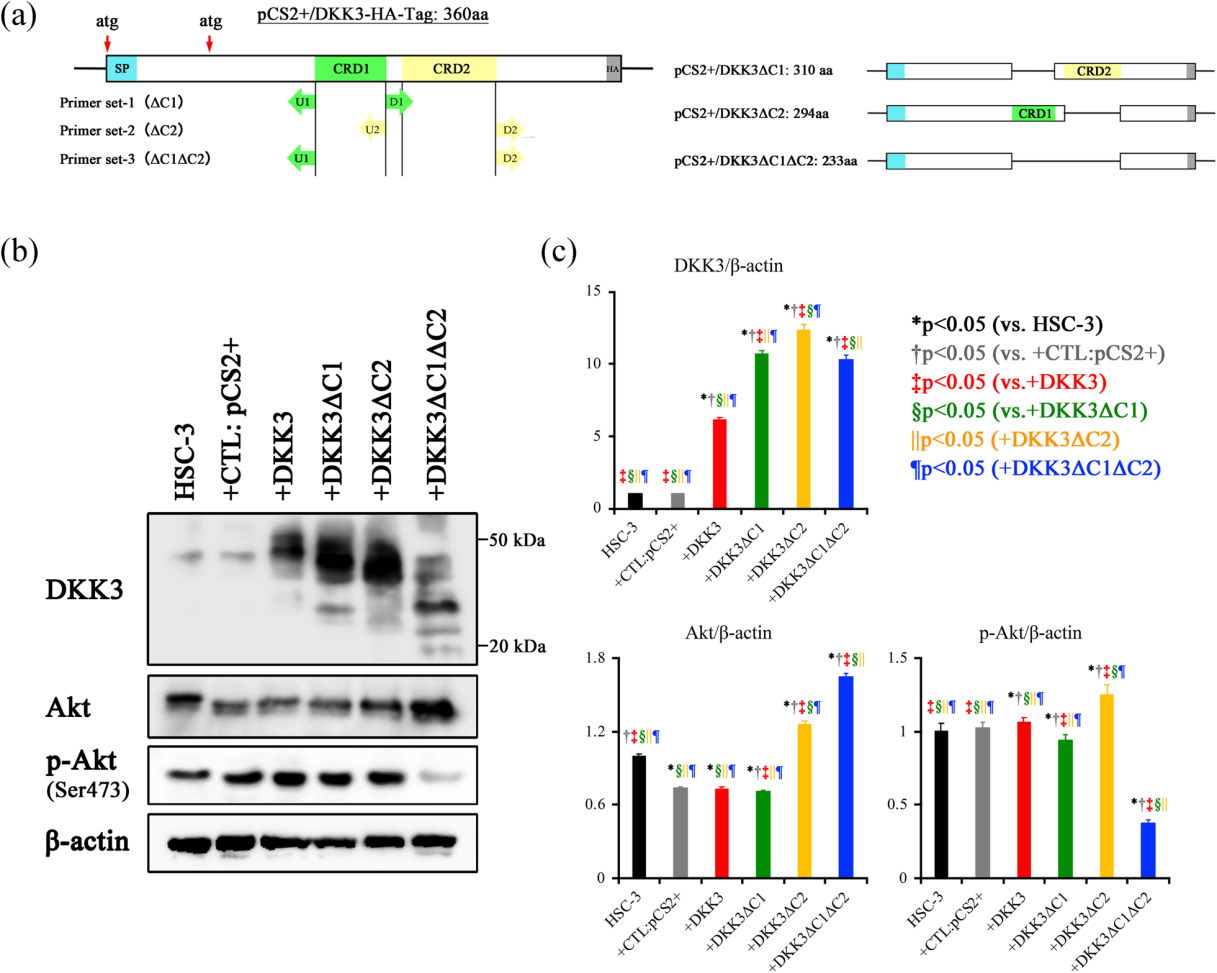
DKK3 deletion mutant expression plasmids and their effect on Akt phosphorylation. **a** Schematic explanation for the generation of DKK3 deletion mutant plasmids; DKK3ΔC1, DKK3ΔC2 and DKK3ΔC1ΔC2. The template DKK3 expression plasmid codes full-length DKK3 protein (consisting of 350 aa) with HA-tag in the COOH-terminal. Inverse PCR with primer ser U1 and D1, U2 and D2, and U1 and D2 resulted in the expression plasmid of DKK3ΔC1 (300 aa), DKK3ΔC2 (284 aa) and DKK3ΔC1ΔC2 (223aa), respectively. atg: start codon, SP: signal peptide, HA: HA-Tag. CRD1: cysteine rich domain1 (DKK-type Cys-1), CRD2: cysteine rich domain 2 (DKK-type Cys-2). (b) Amino acid sequences of DKK3ΔC1, DKK3ΔC2, and DKK3ΔC1ΔC2. **b** DKK3 expression is significantly elevated in DKK3 plasmid and DKK3 deletion mutant plasmids. Note that the band size was changed because of deletion and the band was shifted. **c** Akt expression is significantly elevated in DKK3ΔC2 and DKK3ΔC1ΔC2 transfectants. Phosphorylated Akt (p-Akt) was significantly elevated in DKK3 and DKK3ΔC2 transfectants, while it was significantly decreased in DKK3ΔC1ΔC2 transfectant compared to that in all groups

A DKK3 expression plasmid was generated in our previous study [14]. Using this plasmid as a template, three plasmids with deletion mutants were newly generated by inverse PCR (Fig. 1A). The primer sequences used in this procedure are listed in Table 1. Inverse PCR was performed using the KOD-Plus-Mutagenesis Kit (TOYOBO, Osaka, Japan). The PCR conditions were as follows: 96 ℃ for 1 min, followed by 25 cycles of 96 ℃ for 30 s, 60℃ for 30 s and 68 ℃ for 5 min, and a final extension step at 68 ℃ for 10 min. Then the PCR products were digested with the restriction enzyme Dpn I (New England Biolabs, Inc., Ipswich, MA, USA), followed by the self-ligation and transformation in DH5α competent cells (TOYOBO). DNA was extracted from selected colonies using the QiAprep Spin Miniprep Kit (QIAGEN, Hilden, Germany). After checking the sequences (GENEWIZ, South Plainfield, NJ, USA), the plasmids were amplified and stocked. The plasmids lacking CRD1, CRD2, and both CRD1 and CRD2 were named as DKK3ΔC1, DKK3ΔC2, and DKK3ΔC1ΔC2, respectively.

**Table 1:** Primer sequences for inverse PCR

Primer	Sequence
DKK3DC1-U	5′-CTC GTG GCT CCT TCT GCC TTC TTC GTC-3′
DKK3DC1-D	5′-AAA ATG GCC ACC AGG GGC AGC AAT GGG-3′
DKK3DC2-U	5′-GAT GGT CCC ATT GCT GCC CCT GGT GGC C-3′
DKK3DC2-D	5′-CAG CCC CAC AGC CAC AGC CTG GTG TAT G-3′

The plasmids were transfected into the cells using Turbofectin 8.0 (OriGene Technologies, Inc., Rockville, MD, USA) according to the manufacturer’s instructions. The pCS2^+^ empty vector (Addgene, Cambridge, MA, USA) was used as a control for transfection. Protein expression was examined by western blotting.

### Western blotting (WB)

The cells were maintained until they reached confluence. Then the cell lysate was harvested in IP buffer [20 mM Tris–HCl (pH 8.0), 150 mM NaCl, 1 mM EDTA, 1% Triton X-100]. The amount of protein was quantified using DC^TM^ Protein Assay (Bio-Rad Laboratories, Hercules, CA, USA), and 5 mg of protein was used. The cell extracts were boiled in Laemmli’s buffer for 3 min. Each protein sample was loaded onto an e-PAGEL precast gel (ATTO, Tokyo, Japan) and then blotted onto polyvinylidene difluoride (PVDF) membranes. Following the blockade of nonspecific binding by soaking the PVDF membranes in PVDF Blocking Reagent for Can Get Signal (TOYOBO) at room temperature for 1 h, the membranes were treated with primary antibodies at 4 ℃, overnight. The primary antibodies used in the study were DKK3 (ab186409, Abcam, Cambridge, MA, USA), Akt (4685S, Cell Signaling Technology, Inc., Danvers, MA, USA), p-Akt (Ser473) (9271S, Cell Signaling Technology), and β-actin (5057S, Cell Signaling Technology) at 1:500–1:1,000 dilution. The membranes were then rinsed in TBST, followed by incubation with secondary antibody (Jackson Immuno Reasearch Inc., West Grove, PA, USA) for 1 h at room temperature. The antibodies were diluted in Can Get Signal (TOYOBO). The proteins were visualized using the ECL Prime Western Blotting Detection System (GE Healthcare, Buckinghamshire, UK). Band density was analyzed using ImageJ software version 1.51 (http://rsb.info.nih.gov/ij/; National Institutes of Health, Bethesda, MD, USA).

### Cell proliferation assay

To assess the effects of DKK3 deletion mutants and the complementary peptides on cellular proliferation, MTT assay was performed using the TACS^®^Cell Proliferation Assay kit (Trevigen, Gaithersburg, MD, USA). Cells were seeded into a 96-well microplate at 1.0 × 10^3^ cells/100 mL/well and were cultured for 24 h. MTT reagent was added to the cells and incubated for 4 h at 37 ℃ in an atmosphere containing 5% CO_2_, resulting in the formation of formazan crystals. Subsequently, the detergent agent included in the kit was added, and the absorbance was measured at 570 nm. Data were acquired on days one, three, and five. The data are displayed as relative values, where the data of day one were converted to 1.0.

### Invasion assay

BioCoat^™^ Matrigel^®^ Invasion Chambers (Corning Life Sciences, Bedford, MA, USA) were used to conduct an invasion assay, according to the manufacturer’s protocol. Cells were harvested and suspended in serum-free DMEM at 2.5 × 10^5^ cells/mL; 500 mL cell suspension was added into the upper chambers. After 24 h of incubation at 37 ℃ in an atmosphere containing 5% CO_2_, the chambers were fixed and stained with Diff-Quik Stain (Lab Aids Pty Ltd., North Narrabeen, NSW, Australia) and mounted on a glass slide. The cell number was counted under an optical microscope, and the relative cellular invasion (% invasion) was calculated according to the manufacturer’s protocol.

### Migration assay

The cell migration assay was performed using an Ibidi Culture-insert (Ibidi GmbH, Munich, Germany). Cells were suspended in DMEM supplemented with 10% FBS (1.0 × 10^6^ cells/mL); and 70 mL cell suspension was transferred to each well of the Culture‐insert set on a 6‐well plate. After 24 h of incubation at 37 ℃ in an atmosphere containing 5% CO_2_, the Culture‐insert was removed using sterilized tweezers. Photos were taken immidiately after the removal of the Culture‐insert (0 h) and 6 h later. The area was measured using ImageJ software version 1.51 and the wound healing was calculated.

### Design of complementary peptides for CRDs of DKK3

To design the peptide sequences for interaction with the target regions in the CRDs of DKK3, we used the evolutionary software program MIMETIC (Research Institute for Protein Science Co. Ltd., Nagoya, Japan). This proprietary software employs a genetic algorithm that generates a series of increasingly optimized peptides for a target by random alteration of amino acids for 5000 generations. Each peptide sequence generated in this manner was assigned a score based on several physicochemical parameters. Following the final generation, the program re-arranges the peptides into a list according to a scoring method for “goodness of fit” to the target [16, 17]^.^ We obtained ten complementary peptides sequences against CRDs (20 amino acid (aa) sequences for CRD1 and 18 aa for CRD2, respectively).

### Generation of structure models of DKK3 and peptides, and docking simulation

Before verifying whether the designed peptide actually binds to the CRD of DKK3, we simulated it in silico using computational science technology. First, we created a model of DKK3 and the peptides. Homology modeling (HM) method is commonly used to create the crystal structures of proteins. In the HM method, it is necessary to perform a homology search in the RCSB PDB (http://www.rcsb.org/) [18]; if a matched amino acid sequence exists, models can be created using the matched sequences as a template. After template selection, models were created through target template alignment, model building, and model evaluation [19]^.^ The prediction accuracy depends on the homology of the template. To create the DKK3 model, CRD2 of the mouse dkk2 (PDB ID:2JTK) is commonly used as a template [19–21], but the homology score was not sufficient for our experiment. Moreover, there were no templates for peptides to create models using HM. Therefore, we attempted to create models only from amino acid sequences using de novo or ab initio modeling methods. De novo methods generally require vast computational resources, such as Raptor X (http://raptorx.uchicago.edu/), which is a web server that employs a powerful in-house deep-learning model and can predicts the structural properties of a protein sequence without using any templates [22–27]. To model the peptide, Raptor X requires amino acid sequences longer than 26 aa residues, and oligoarginine (RRRRRRRRRRRR) was added to the complementary peptide sequence. This confers the peptide with high efficiency of internalization and facilitates intracellular delivery of the peptide [28–30], and also fulfills the need for experiments to inhibit both secreted DKK3 and the non-secreted, intracellular isoform of DKK3 at the same time. Finally, we obtained structural models of DKK3 and the peptides. All the models were confirmed using UCSF Chimera software (https://www.rbvi.ucsf.edu/chimera) [31]. Potein-peptide docking was performed using ClusPro2 (https://cluspro.bu.edu/) [32–35]. The model scores were evaluated using the lowest energy of coefficient weighted score with balanced coefficients. We tested two peptide sets: the set with the best score (peptides for CRD1-#6 and CRD2-#5) and the set with the worst score (peptides for CRD1-#1 and CRD2-#1). The data for the former are shown in Figs. 4, 5, 6 and 7 and those of the latter are shown in Additional file 1:Fig. S1, Additional file 2: Fig. S2, Additional file 3: Fig. S3.

**Table 2:** Amino acid sequence of the complementary peptides

Peptide	Sequence	Lowest energy score*
Peptides for CRD1 + oligoarginine (Rn = 12)		
#1	ANTVTIKLSASDMTVSDLETRRRRRRRRRRRR	− 1351.6
#2	ANTVTITATATTTATTTLHTRRRRRRRRRRRR	− 1390.2
#3	AVTVTKWLTATTTATTTLHTRRRRRRRRRRRR	− 1630.1
#4	AVTVTTQASALTTMTSETLTRRRRRRRRRRRR	− 1480.9
#5	AVTVTTVATATTMTTTSMMTRRRRRRRRRRRR	− 1506.1
#6	AVTVTWTLTATTTATTTLHTRRRRRRRRRRRR	− 1636.6
#7	TNAVTLQLSTSTTATTLTQTRRRRRRRRRRRR	− 1408.9
#8	TVTPASITCTTSTATTTLQTRRRRRRRRRRRR	− 1464.3
#9	TVTVAQTATTLSTATTSLKTRRRRRRRRRRRR	− 1436.0
#10	TVTVATPTTALSTATSTLTYRRRRRRRRRRRR	− 1481.5
Peptides for CRD2 + oligoarginine (Rn = 12)		
#1	AFPRQPPQFPPPTLTLKKRRRRRRRRRRRR	− 1420.8
#2	AFRPQYYPTFQTQLTLPKRRRRRRRRRRRR	− 517.5
#3	AFRQPPPTYFKWQLSLEWRRRRRRRRRRRR	− 1617.8
#4	AFRQPPQPFWYWELSFEWRRRRRRRRRRRR	− 1623.3
#5	APFQRYWQFTYWKLSLEWRRRRRRRRRRRR	− 1922.7
#6	AWYQPPPQFTYWKLSLEWRRRRRRRRRRRR	− 1673.6
#7	AYQWYWYQPFQTTLTLEKRRRRRRRRRRRR	− 1518.6
#8	AYRFQPPQPFQTTLTLEKRRRRRRRRRRRR	− 1580.2
#9	AYRQFPPPKFYWDLSLERRRRRRRRRRRRR	− 1569.9
#10	AYRQFPPPTYQPTLAVKRRRRRRRRRRRRR	− 1478.2

^*^Lowest energy in balanced coefficient weights

### Peptide synthesis

Based on the docking simulation data, we synthesized the complementary peptides (GenScript Japan Inc., Tokyo, Japan). The peptides were applied to the HSC-3 cells under the following condition; 10 nM, 25 nM, 50 nM, 100 nM, and 500 nM, and the suppressive effect of Akt phosphorylation was assessed by western blotting. The bands of p-Akt were normalized with β-actin, a regression formula was generated using Image J, and finally half maximal (50%) inhibitory concentration (IC_50_) was calculated. Based on these results, peptides with the best and the worst scores were added at 100 nM and 500 nM, respectively, for the following experiments.

### Xenograft model and histological evaluation

Cells were suspended in phosphate-buffered saline (PBS) at 5.0 × 10^6^ cells/150 µL and were subcutaneously injected into the dorsal area of five-week-old, male BALB/cAJcl-nu/nu nude mice (CLEA Japan, Inc., Tokyo, Japan). The animals had free access to food and water and were housed at 25 °C (60–70% humidity), under a 12 h light/dark cycle.

Twelve mice were used in this study, which were divided into two groups: (i) injected with HSC-3 cells and (ii) HSC-3 + peptide. The tumor volume (V) was measured and calculated using the following formula: ${\text{V}}\, = \,{4}/{3}\pi \, \times \,{\text{L}}/{2}\, \times \,\left( {{\text{W}}/{2}} \right)^{{2}}$, where L is the longest diameter and W is the diameter perpendicular to L.

The tumor mass was formed on day 14 and then gradually increased in size. On day21, peptides were injected in 100 nM or 500 nM per tumor volume 100 mm^3^. The peptides or control (solvent, PBS) were injected on days 21, 24, 28, and 31, and the tumor volume was measured on days 21, 23, 25, 28, 30, and 32. On day 35, the mice were sacrificed, and the tumors were collected for histological evaluation. The experimental schedule is shown in Fig. 7a. This study was performed in accordance with the Guidelines for Animal Experiments at Nagasaki University, and the animal protocol for this study was approved by the Animal Care and Use Committee of Nagasaki University (no. 210922-1, 2021).

Tissues were then fixed in 10% neutral buffered formalin for 8 h at room temperature, embedded in paraffin, sectioned at 4 μm, and stained with hematoxylin–eosin (HE). In addition, the tissue sections were subjected to immunohistochemistry (IHC). IHC for Ki-67 was performed using VENTANA Bench Mark ULTRA (VENTANA, Tucson, AZ, USA), and antibody against Ki-67 (30-9, VENTANA), according to the manufacturer’s instructions. Ki-67 positive cells were counted, and the Ki-67 labeling index was calculated.

### Statistical analyses

All values are presented as the means ± standard deviation. Significant differences were determined using a two-tailed Student’s t-test with Bonferroni correction. All analyses were performed using R version 3.5.1 (http://www.r-project.org/). A P-value of p < 0.05 was considered to be statistically significant.

## Results

### Transfection of DKK3 deletion mutant and its effects on Akt activation

Transfection of full-length of DKK3 or DKK3 deletion mutants was assessed using western blotting. As expected, transfection with an empty vector (CTL: pCS2^+^) did not affect the expression of DKK3 and phosphorylation of Akt, and the full-length DKK3 transfectant showed significantly increased DKK3 expression and Akt phosphorylation. All the transfectants of DKK3 deletion mutants showed elevated DKK3 expression with shifted band size, while phosphorylation of Akt significantly decreased only in DKK3DC1DC2 transfectants (Fig. 1b, c).

### Transfection of DKK3 deletion mutant and its effects on malignant potentials

**Fig. 2:**
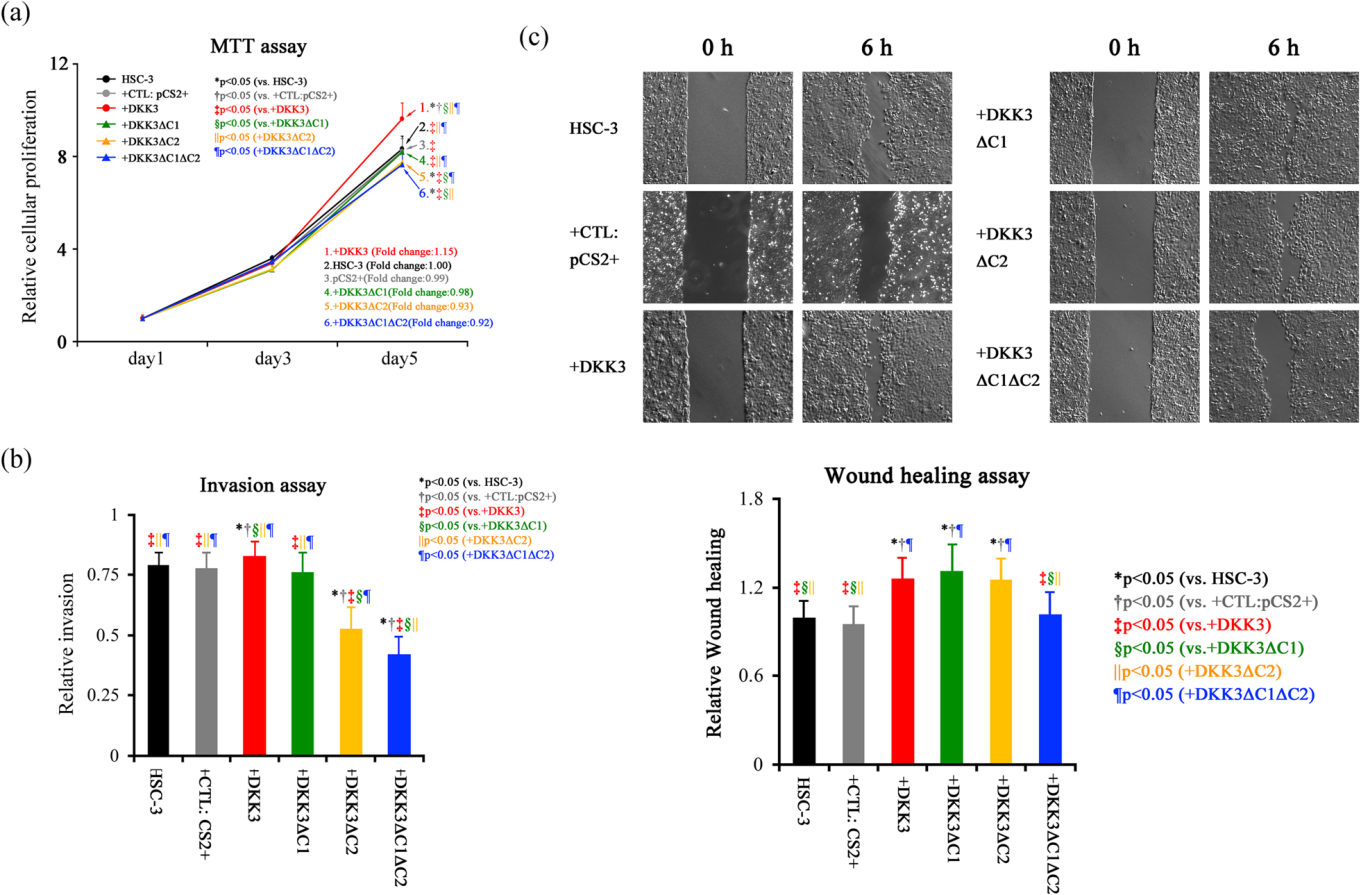
DKK3 deletion mutant expression plasmids and their effect on cellular proliferation, invasion, and migration. **a** MTT assay revealed that DKK3 transfection resulted in significantly increased cellular proliferation, while that of DKK3ΔC1 transfectant or DKK3ΔC2 transfectant was quelled to the control level and that of DKK3ΔC1ΔC2 was significantly diminished compared to all group. **b** Cancer cell invasion was significantly elevated in DKK3 transfectants, whereas DKK3 ΔC1 is not. Transfection with DKK3ΔC2 and DKK3ΔC1ΔC2 significantly decreased cancer cell invasion. **c** The wound healing assay showed that transfection of DKK3, DKK3ΔC1 and DKK3ΔC2 significantly elevated cancer cell migration, and transfection of DKK3ΔC1ΔC2 showed suppressed cellular migration to the control level

Transfection of full length DKK3 resulted in significantly elevated cellular proliferation (fold change 1.15, compared to HSC-3), and transfection of deletion mutants was suppressed. In particular, DKK3DC1DC2 transfectants showed a significantly decreased compared to any other groups (fold change 0.92, compared to HSC-3) (Fig. 2a). Invasion assay revealed that full-length DKK3 significantly elevated cellular invasion, which was significantly decreased in DKK3ΔC2 transfectants and DKK3ΔC1ΔC2 transfectant, while transfection of DKK3ΔC1 did not affect invasion (Fig. 2b). Cellular migration was significantly elevated in cells transfected with full-length DKK3, DKK3ΔC1, and DKK3ΔC2, whereas migration of DKK3ΔC1ΔC2 transfectants was suppressed to the control level (Fig. 2c).

### Detection of the specific site of the functional domain and generation of DKK3 complementary peptides

**Fig. 3:**
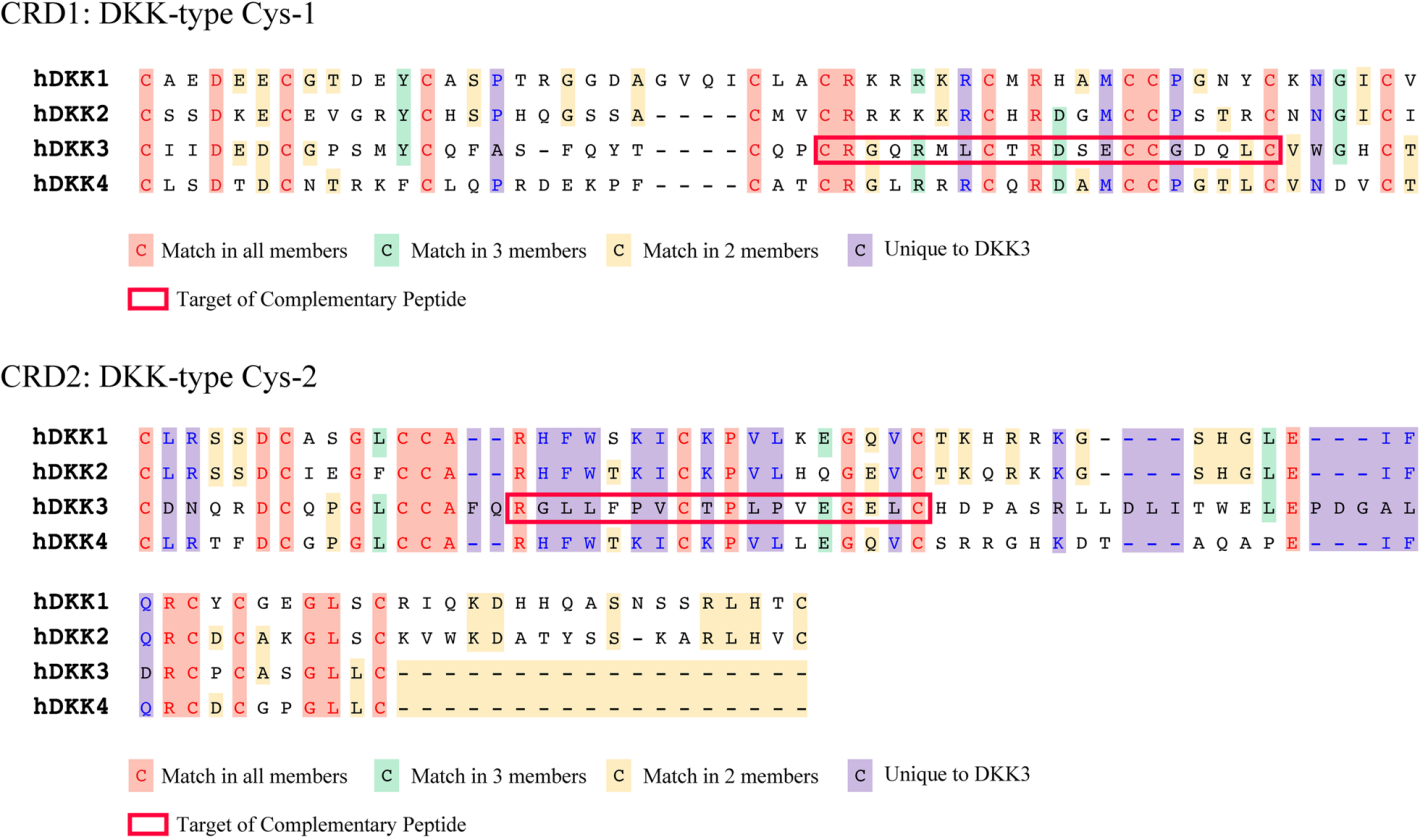
Identification of specific amino acid sequences of DKK3 cysteine rich domains. Comparing the amino acid sequences of CRD1 and CRD2 in human DKK family members, we hypothesized that the region that containing enriched unique amino acid residues within the CRD might be of great importance for exerting of oncogenic function. The sequence surrounded by the red frame was chosen as the target for complementary peptides

Based on these data, we hypothesized that both CRD1 and CRD2 will act as functional domains and are important for the binding of effector proteins. We then compared the amino acid sequences of CRDs among the DKK family members, and defined the core sequences based on the amino acid sequences that contains unique amino acids in DKK3. The putative core amino acid sequences for CRD1 and CRD2 were CRGQRMLCTRDSECCGDQLS and RGLLFPVCTPLPVEGELC, respectively (Fig. 3).

**Fig. 4:**
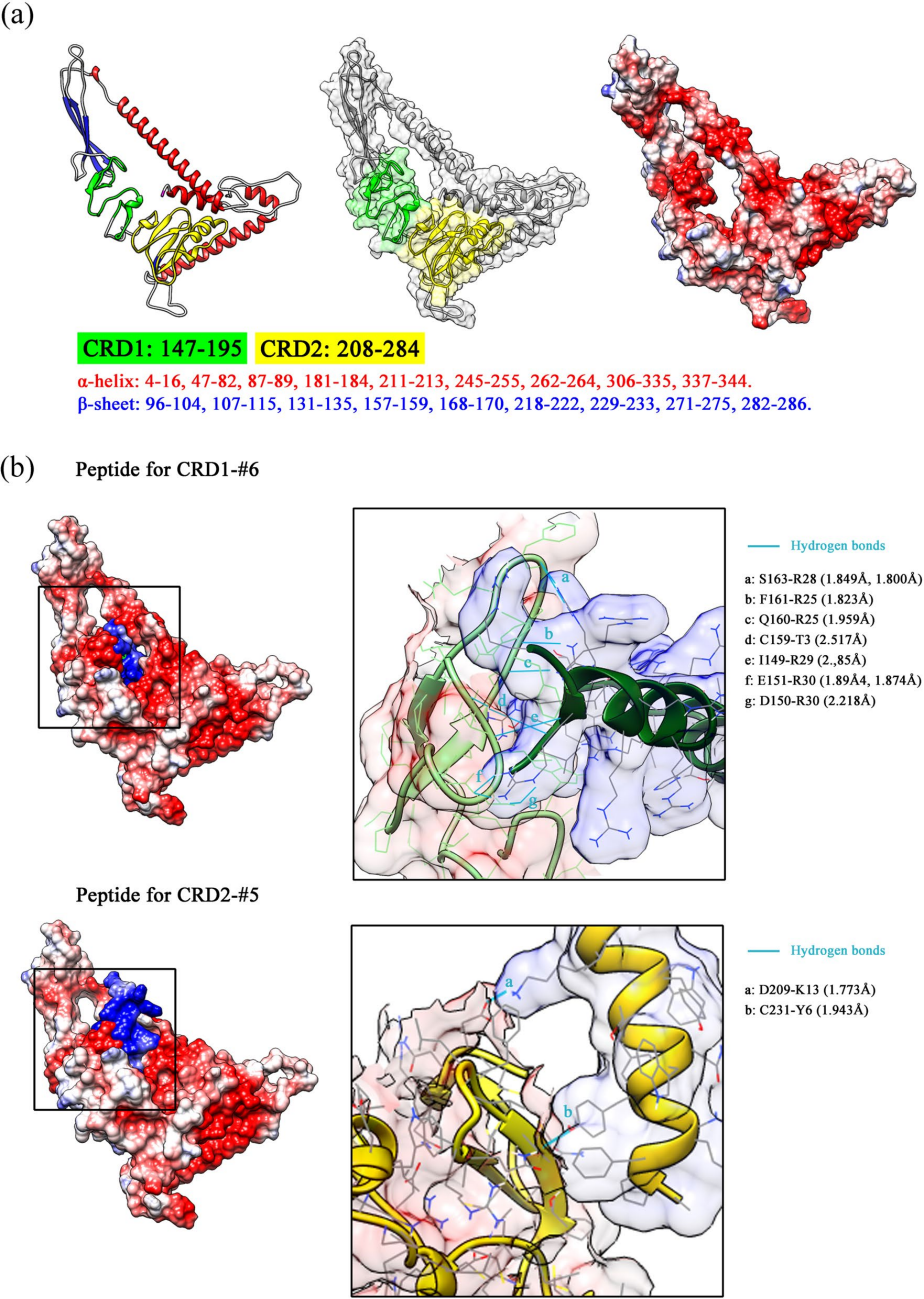
Models of DKK3 and peptides, and docking simulation. **a** The model of DKK3. The predicted structure; α-helix, β-sheet, CRD1, and CRD2, are colored in green, yellow, red, and blue, respectively. The number in α-helix, β-sheet means the corresponding amino acid (left). The surface was displayed in the model (center), together with the structures as ribbon (right). **b** Docking simulation results with the lowest energy are shown; #6 for CRD1 and #5 for CRD5. The enlarged illustration in the black frame shows the predicted binding sites

We used the evolutionary software program MIMETIC to obtained ten amino acid sequences for the complementary peptides (shown in Table 2). Then we generated the models for full-length DKK3 protein and peptides using Raptor X (Fig. 4a), and a docking simulation was performed using ClusPro2. Based on the lowest energy score, we chose peptides for CRD1-#6 and CRD2-#5. The results of the docking simulations are shown in Fig. 4b.

### Effects of DKK3 complementary peptides on Akt and malignant potentials of the cancer cells

**Fig. 5:**
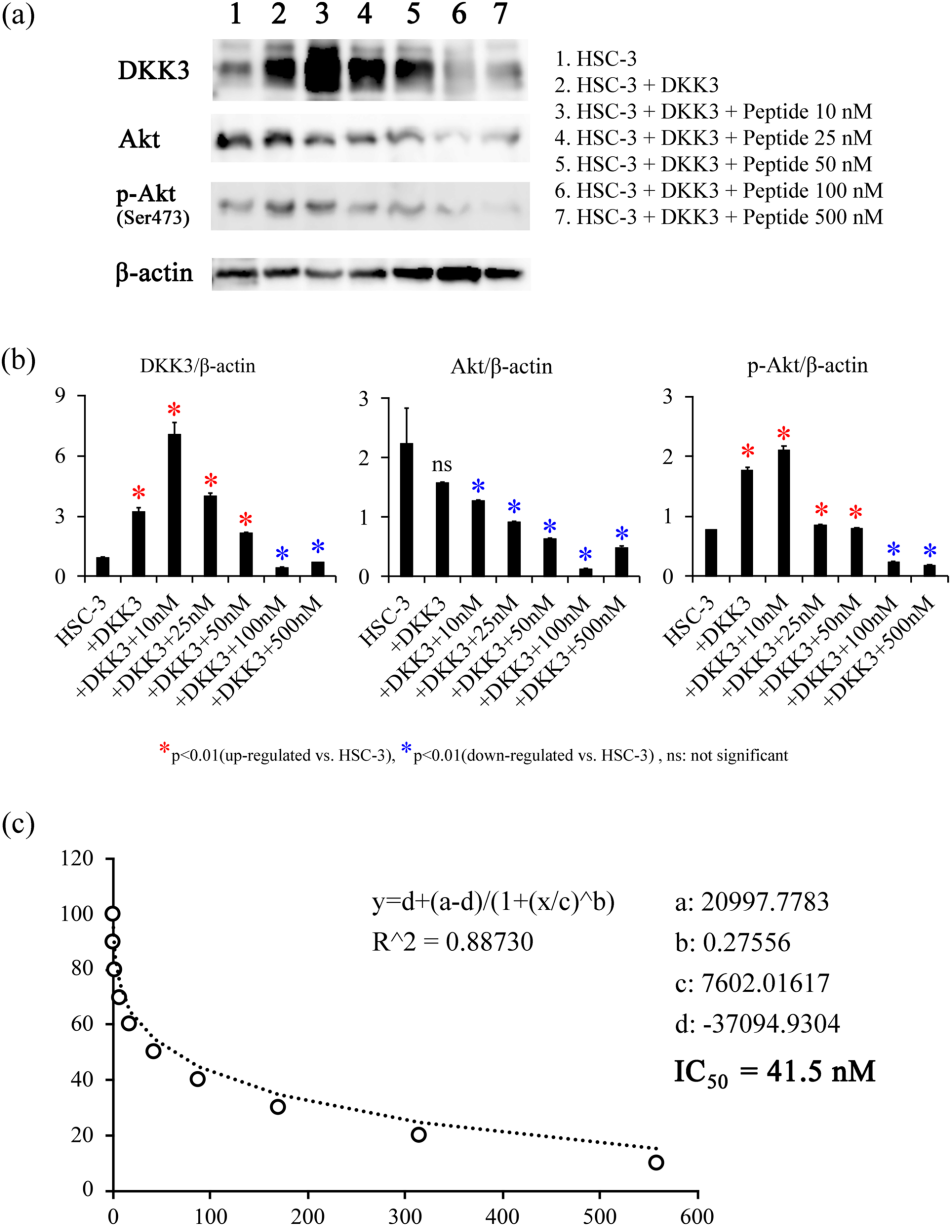
Effects of the peptides on Akt phosphorylation. **a** Transfection of full-length DKK3 resulted in an elevated expression of DKK3, and administration of peptide reduced the phosphorylation of Akt, together with DKK3 expression in a dose-dependent manner. **b** Phosphorylation of Akt was elevated when full-length DKK3 was transfected, which was significantly suppressed by peptide. **c** The IC_50_ for suppression of phosphor-Akt was calculated as 41.5 nM

Western blotting revealed that the addition of both complementary peptides (CRD1-#6 and CRD2-#5) simultaneously decreased Akt phosphorylation in a dose-dependent manner (Fig. 5a, b), and IC_50_ was calculated as 41.5 nM (Fig. 5c). We then added 100 nM of the peptides into the cells and assessed their effects on cellular proliferation, migration, and invasion.

**Fig. 6:**
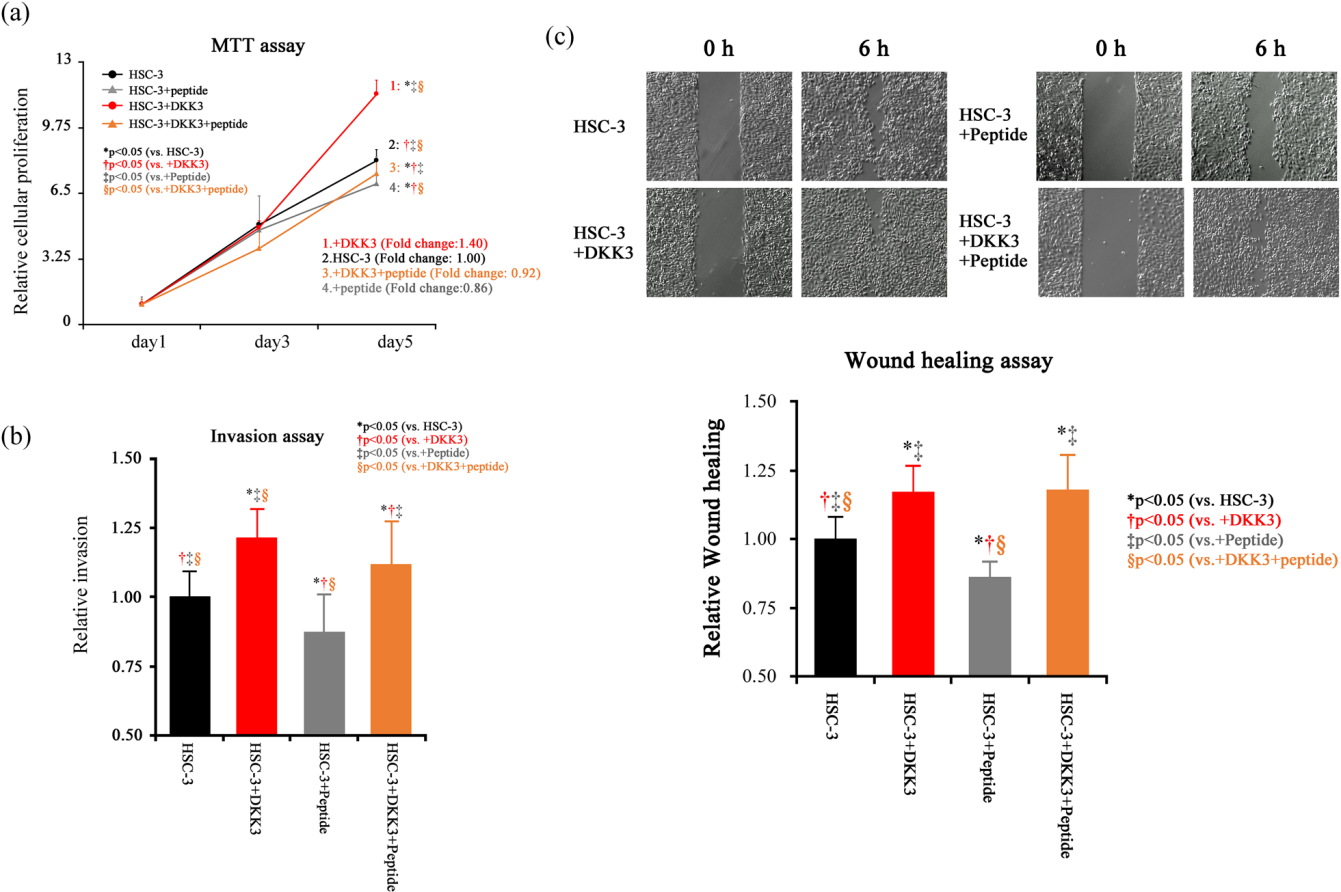
The effects of the peptides on cellular proliferation, invasion and migration. **a**–**c** DKK3 overexpression significantly elevated cellular proliferation, invasion, and migration. The administration of peptides significantly suppresses cellular proliferation, invasion, and migration, and cancels the elevating effects of DKK3 transfection on them

The peptides significantly suppressed cellular proliferation and invasion, and cancelled the augmentative effect of full-length DKK3 transfection (Fig. 6a, b). As for cellular migration, peptides could significantly suppressed the cellular migration, but this was insufficient to cancel the augmentative effect of full-length DKK3 transfection (Fig. 6c).

### Effects of DKK3 complementary peptides in the xenograft model

**Fig. 7:**
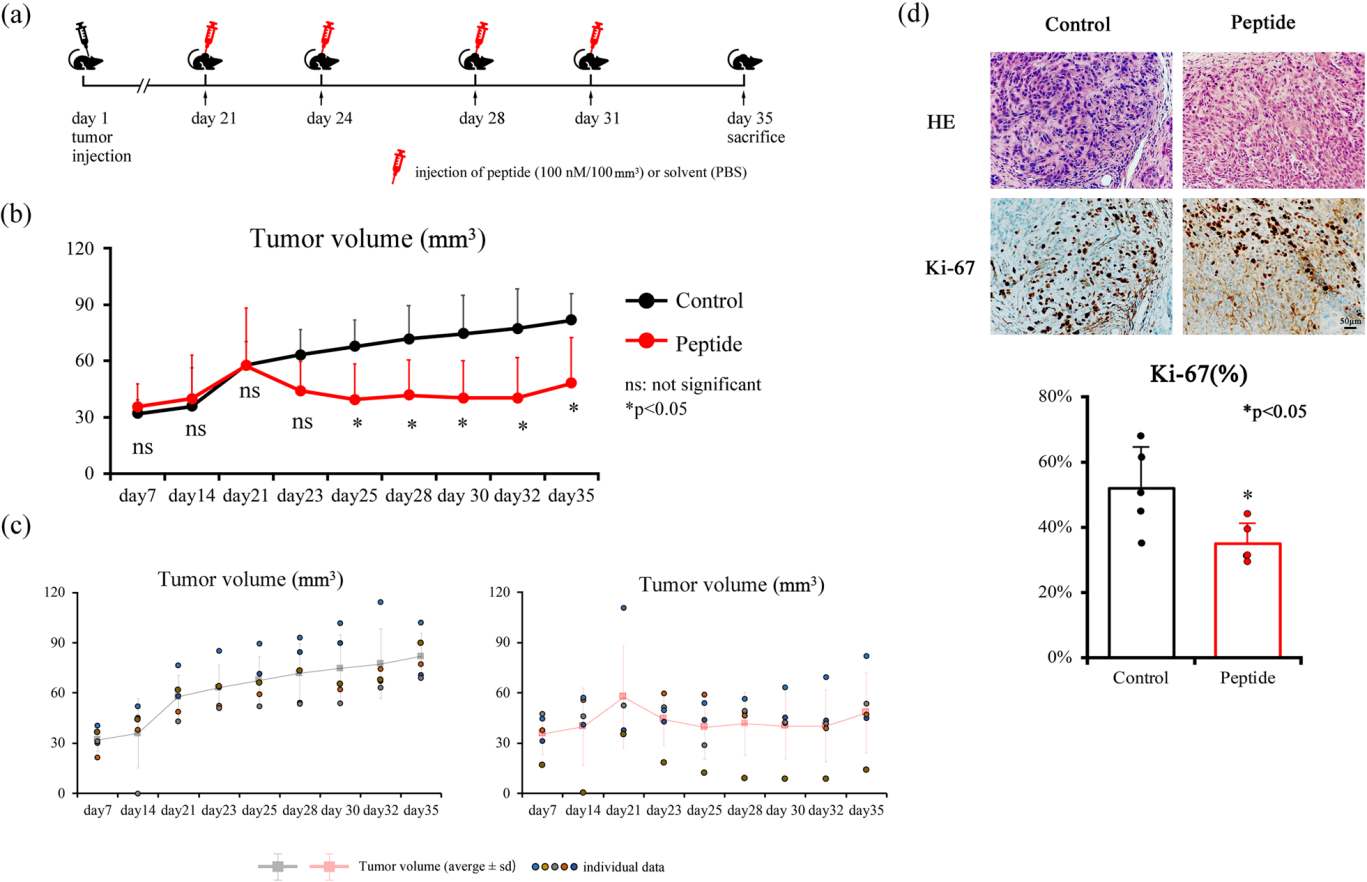
The therapeutic effect of the peptides in the xenograft model. **a** Schematic explanation for the schedule of the animal experiments. **b** The tumor volume was significantly reduced by the administration of peptide from days 25 to 35. **c** The tumor volume of the individual mice is shown. The average ± standard deviation (sd) is also displayed. **d** There were no histological differences between the control group and the peptide groups, but the Ki-67 index of the peptide group was significantly decreased compared to the control group

In this model, a tumor mass was formed on day14. We started the treatment from day 21, as all the tumor sizes reached to > 50 mm^3^. The mean tumor size of the control and peptide group on day 21 were 57.68 ± 12.85 mm^3^ and 57.50 ± 30.70 mm^3^, respectively. Administration of the peptide on day 21 resulted in reduced tumor size on day 23, but the difference was not statistically significant (p = 0.074). Consecutive peptide administration suppressed tumor growth, and significant differences were observed on days 25 (p = 0.028), 28 (p = 0.031), 30 (p = 0.027), 32 (p = 0.025), and 35 (p = 0.027). The final tumor size of the control group and peptide groups on day 35 were 81.79 ± 13.97 mm^3^ and 48.12 ± 24.19 mm^3^, respectively (Fig. 7b).

Histological evaluation of the tumors revealed that there were no histological differences between the control group and peptide groups. However, the Ki-67 index of the peptide group was significantly lower than that of the control group (p = 0.032) (Fig. 7c).

## Discussion

HNSCC accounts for approximately 90% of malignancies in the head and neck region, and despite the advances in diagnosis, imaging, treatment modalities, and cancer genetics, its five-year overall survival has not substantially improved over the last few decades [36]. New therapeutic strategies that enable less-invasive treatment of HNSCC are required, and to make it a reality, it is necessary to understand the molecular background of HNSCC. Although development in next-generation sequencing has uncovered some aspects of malignant tumors, key genes or pathways that could be a draggable target have not been identified yet. In this context, we attempted to identify HNSCC-specific cancer-associated genes focusing on DKK3.

DKK3 belongs to the Dickkopf WNT signaling pathway inhibitor family (DKK family), consisting of DKK1, DKK2, DKK3, and DKK4 and encodes two distinct CRDs. DKK family members are reported to function as tumor suppressors, because of their inhibitory function in the oncogenic Wnt signaling pathway [7, 8, 37, 38]. DKK3 is also known as Reduced Expression in Immortalized Cells (REIC), whose expression is reduced in many kinds of malignancies, because of promoter hypermethylation [39–41]. It has been reported that the over-expression of REIC/DKK3 in cancer cells can induce apoptosis, and there are clinical trials of REIC/DKK3 gene therapy for prostate cancer [42], liver cancer [43], and endometrial cancer [44]. Admittedly, DKK3 functions as tumor suppressor and loss of DKK3 function is associated with poorer outcomes in DKK3 non-expressing tumors, such as prostate cancer and renal cancers, which is also confirmed by the analyses using by data in TCGA [15].

However, some studies have demonstrated that DKK3 expression does not always decrease in cancer, and its function is context-dependent [13]. Our previous studies have demonstrated that: (1) DKK3 expression is decreased in many kinds of malignancies, but it is specifically high in squamous cell carcinoma of the head and neck, esophagus, and pancreatic ductal adenocarcinoma. (2) DKK3 has been reported to be a tumor suppressor, and its forced overexpression by adenovirus causes cancer cell apoptosis. However, DKK3 over-expression did not induce apoptosis in HNSCC cells [14]. (3) DKK3 expression is also high in HNSCC tissue samples, and DKK3 expression is correlated with poorer overall survival and is an independent prognostic marker [11, 13]. (4) Transitional knockdown of DKK3 by siRNA could suppressed tumor cell proliferation and migration in HNSCC cells [12]. These findings demonstrate the roles of DKK3. In fact, DKK3 protein expression is high in HNSCC, and DKK3 exerts oncogenic function via activation of Akt, and inhibition of DKK3 or its receptor, cytoskeleton-associated protein 4 (CKAP4), by antibodies resulted in significantly decreased cellular proliferation, migration, and invasion [10–15]. Supporting our results, the oncogenic function of DKK3 has been reported in DKK3-expressing cancers, including pancreatic ductal adenocarcinoma (PDAC), esophageal squamous cell carcinoma (ESCC), and esophageal adenocarcinomas (EAC). Briefly, PDAC expresses DKK3, and its knockdown significantly reduced cellular proliferation [45], and treatment with a DKK3-blocking monoclonal antibody inhibited the PDAC progression and chemoresistance and prolonged survival in PDAC cell xenograft model [46]. DKK3 knockdown attenuated ESCC-derived cancer cell proliferation and significantly suppressed the ESCC-derived cell growth in vivo [47]. In EAC, DKK3 is overexpressed and is associated with a high frequency of nodal metastasis, and overexpression of DKK3 results in significantly increased cellular proliferation and invasion [48]. Taken together, DKK3 may determine oncogenic properties in DKK3-expressing cancers; therefore, it is a promising therapeutic target for such cancers.

In the present study, we identified the functional domain of DKK3. This is because DKK3 might activate Akt via two pathways: secreted DKK3 and via non-secreted DKK3 [15]. Furthermore, while inhibition of DKK3 by the antibody was effective enough, but the effects differed among cell lines [13]. Considering the fact that actual HNSCC tissue includes a heterogeneous cellular population, blockade of the both of pathways targeting protein–protein interactions (PPIs) is thought to be more reasonable.

To inhibit PPIs, we needed to identify the functional domain of DKK3. There are several reports on the PPIs of DKK family members. DKK family members have two distinct CRDs (CRD1 and CRD2), which are involved in the functional domain. So far, it has been reported that DKK family members may interact with LRP6, Kremen 1/2 [37]. Because of the structural divergence in DKK3, the overall protein sequence homology between DKK3 and other DKK members is less than 40%, whereas those of DKK1, DKK2, and DKK4 is approximately 50% [37, 38, 49, 50]. The structures predicted by AlphaFold revealed that the structures of DKK1, DKK2, and DKK4 are similar, but DKK3 is strikingly different from those of other DKK members, especially in CRD2 [49, 51]. CRD2 is associated with the binding of DKKs (DKK1, DKK2, and DKK4) to LRP6 or Kremen1/2, however, DKK3 can bind neither LRP6 nor Kremen1/2 [49, 52, 53]. In this regard, some reports have confirmed whether DKK3 can bind to LRP5/6 or Kremen1/2 using the crystal structures created by the homology modeling (HM) method with CRD2 of the mouse dkk2 (PDB ID:2JTK) as a template [19–21]. However, the results are controversial. Fujii et al. concluded that DKK3 lacks the affinity for LRP5/6 because of insertion of seven amino acids in CRD2 and the existence of P258 [20], while Poorebrahim et al. reported CRD2 is actively involved in the DKK3-LRP5/6 interaction [19].

As for CRD1, it has been reported that CKAP4 binds to all members of the DKK protein via CRD1 [47], and CRD1 of DKK3 is also associated with the binding to transforming growth factor-beta-induced protein (TGFBI) [54]. Another DKK3-binding protein is the β-transducin repeat containing protein (βTrCP) [8], and its binding to DKK3 has been proven by yeast two-hybrid analysis and co-immunoprecipitation [55], although its binding site has not been determined. Moreover, it has also been indicated that both CRD1 and CRD2 are essential for exerting function [56]. We hypothesized that CRD1 and CRD2 are candidate functional domains. We then attempted to generate and transfect the deletion mutants of these domains.

Transfection of deletion mutants (DKK3ΔC1, DKK3ΔC2, and DKK3ΔC1ΔC2) showed that the simultaneous inhibition of both CRD1 and CRD2 at the same time is necessary to inhibit DKK3-driven Akt phosphorylation and oncogenic functions. Next, we sought the amino acid sequences that are essential for these domains to exert their functions. Comparing the amino acid sequences, we found quite unique sequences within CRD1 and CRD2 and designed antisense peptides. Then, the models for DKK3 and peptides were modeled de novo, and after supercomputer-based docking simulation, candidate peptides were generated and verified.

Therapeutic peptides are a novel and promising approach for the development of anti-cancer agents [57]. It has several advantages over monoclonal antibodies: it is relatively inexpensive and easy to synthesize, small in size, and easy to stabilize, store, and transport [58]. In this study, the peptides successfully induced a significant reduction in DKK3-driven Akt phosphorylation, cellular proliferation, migration, and in vivo tumor growth. Notably, this tumor-inhibitory effect was effectuated at a very low dosage, as low as 100 nM. Although the methods for detecting amino acid sequences that would be the core should be improved, and we cannot say that these amino acid sequences are optimized, we believe that the concept of establishing of peptide-based antitumor drug is novel and that we manage to successfully demonstrate its feasibility for the first time.

These results strongly suggests that this peptide will be a prospective therapeutic reagent for HNSCC, and perhaps also for other DKK3-expressing cancers in the near future. The peptide we established targets specifically targets PPIs between DKK3 and its effector proteins, and the mechanism of the action is different from that of the conventional anticancer agents. Both peptide monotherapy and combination therapy of the peptides with other anti-cancer drug are considered as the potential applications of the peptides. However, optimization of the peptide selection and validation for the dose and drug delivery are needed. DKK3 expression would increase in the precancerous lesion, epithelial dysplasia [10], and it has been suggested that increased DKK3 expression occurs in the early stage of the carcinogenesis step of squamous epithelia in the head and neck region. Prevention of the progression of epithelial dysplasia into cancer might be one of the possible applications of these peptides.

We also noted that the inhibition of proliferation by the peptide seemed to not be striking compared to Akt phosphorylation inhibition. This may be because the cellular proliferation of the HNSCC cells is driven by Akt as well as other signaling pathways such as epidermal growth factor receptor (EGFR) [59] and signal transducer and activator of transcription 3 (STAT3) [60]. Moreover, Akt is activated by the mammalian target of rapamycin (mTOR), which is activated by certain signaling molecules other than DKK3. The peptides were then used in combination with conventional (5-fluorouracil or cisplatin) and/or other anticancer agents such as cetuximab (a monoclonal antibody against EGFR), an inhibitor of STAT3 or mTOR.

## Conclusions

In conclusion, we have established a DKK3 specific inhibitory peptide that can suppress tumor proliferation, migration, and invasion at low doses. We will continue to investigate the application of this peptide in a clinical setting.

## Supplementary Information

**Additional file 1: Figure S1.** Effects of peptides with the worst score (peptide2) on Akt phosphorylation. (a) Transfection of full-length DKK3 resulted in an elevated expression of DKK3, and administration of peptide2 reduced the phosphorylation of Akt and DKK3 expression in a dose-dependent manner. (b) Phosphorylation of Akt was elevated when full-length DKK3 was transfected and was significantly suppressed by the peptide. (c) The IC_50_ for suppression of phospho-Akt was calculated as 217 nM.

**Additional file 2: Figure S2.** The effects of peptide2 on cellular proliferation, invasion, and migration. a–c DKK3 over-expression significantly elevated cellular proliferation, invasion, and migration. Administration of peptide2 (500 nM) significantly suppressed cellular proliferation, invasion, and migration and canceled the elevating effects of DKK3 transfection on them.

**Additional file 3: Figure S3.** The therapeutic effect of peptide2 in the xenograft model. (a) Schematic explanation for the schedule of the animal experiments. (b) The tumor volume was significantly reduced by the administration of peptide from days 25 to 35. (c) The tumor volume of the individual mice is shown. The average ± standard deviation (sd) is also displayed. (d) There were no histological differences between the control and peptide groups, but the Ki-67 index of the peptide group was significantly decreased compared to the control group.

## Data Availability

The datasets used and/or analyzed during the current study are available from the corresponding author on reasonable request.

## Abbreviations

Akt: Akt serine/threonine kinase
βTrCP: β-Transducin repeat containing protein
CCND1: Cyclin D1
CDKN2A: Cyclin dependent kinase inhibitor 2A
CKAP4: Cytoskeleton Associated Protein 4
CRD: Cysteine rich domain
CTL: Control
DKK3: Dickkopf WNT Signaling Pathway Inhibitor 3
DMEM: Dulbecco’s modified Eagle's medium
EAC: Esophageal adenocarcinomas
EDTA: Ethylenediaminetetraacetic acid
EGFR: Epidermal growth factor receptor
ESCC: Esophageal squamous cell carcinoma
FBS: Fetal bovine serum
HM: Homology modeling
HNSCC: Head and neck squamous cell carcinoma
HPV: Human papilloma virus
IC_50_: Half maximal (50%) inhibitory concentration
IP: Immunoprecipiration
LRP5/6: Low-density lipoprotein receptor-related protein 5/6
mTOR: Mammalian target of rapamycin
PCR: Polymerase chain reaction
PDAC: Pancreatic ductal adenocarcinoma
PI3KCA: Phosphatidylinositol 3-kinase
PPIs: Protein–protein interactions
PVDF: Polyvinylidene difluoride
REIC: Reduced Expression in Immortalized Cells
STAT3: Signal transducer and activator of transcription 3
TCGA: The Cancer Genome Atlas
TGFBI: Transforming growth factor-beta-induced protein
WB: Western blotting
Wnt: Wingless-type MMTV integration site family
