# Supplementary figures and images for: Establishment of anti-DKK3 peptide for the cancer control in head and neck squamous cell carcinoma (HNSCC)
Source: Cancer Cell Int. 2022 Nov 15;22:352. doi: 10.1186/s12935-022-02783-9 (PMC9664703; doi:10.1186/s12935-022-02783-9)

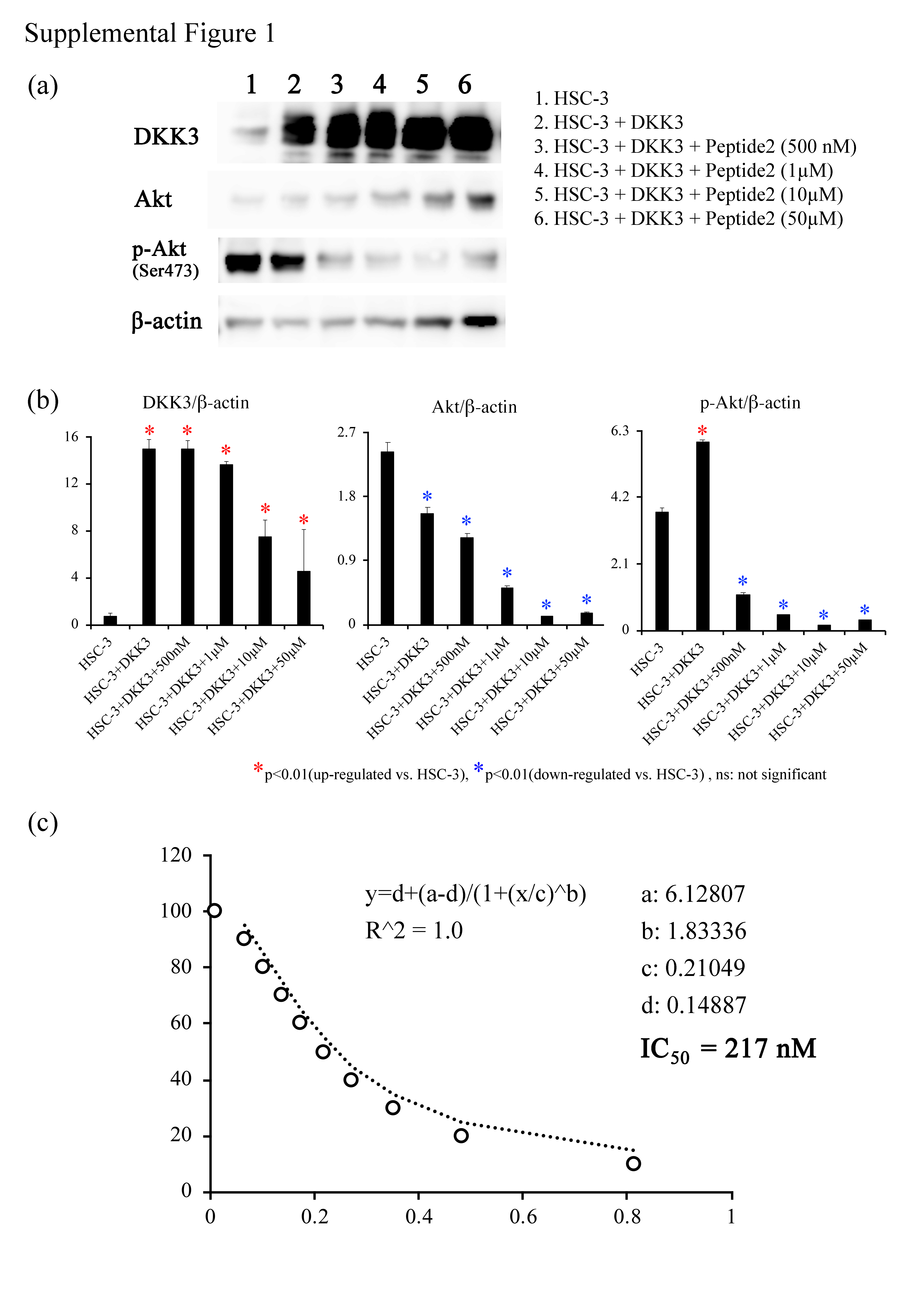

Supplement: Supplementary file 1 — Additional file 1: Figure S1. Effects of peptides with the worst score (peptide2) on Akt phosphorylation. (a) Transfection of full-length DKK3 resulted in an elevated expression of DKK3, and administration of peptide2 reduced the phosphorylation of Akt and DKK3 expression in a dose-dependent manner. (b) Phosphorylation of Akt was elevated when full-length DKK3 was transfected and was significantly suppressed by the peptide. (c) The IC50 for suppression of phospho-Akt was calculated as 217 nM. [file 12935_2022_2783_MOESM1_ESM.jpg]

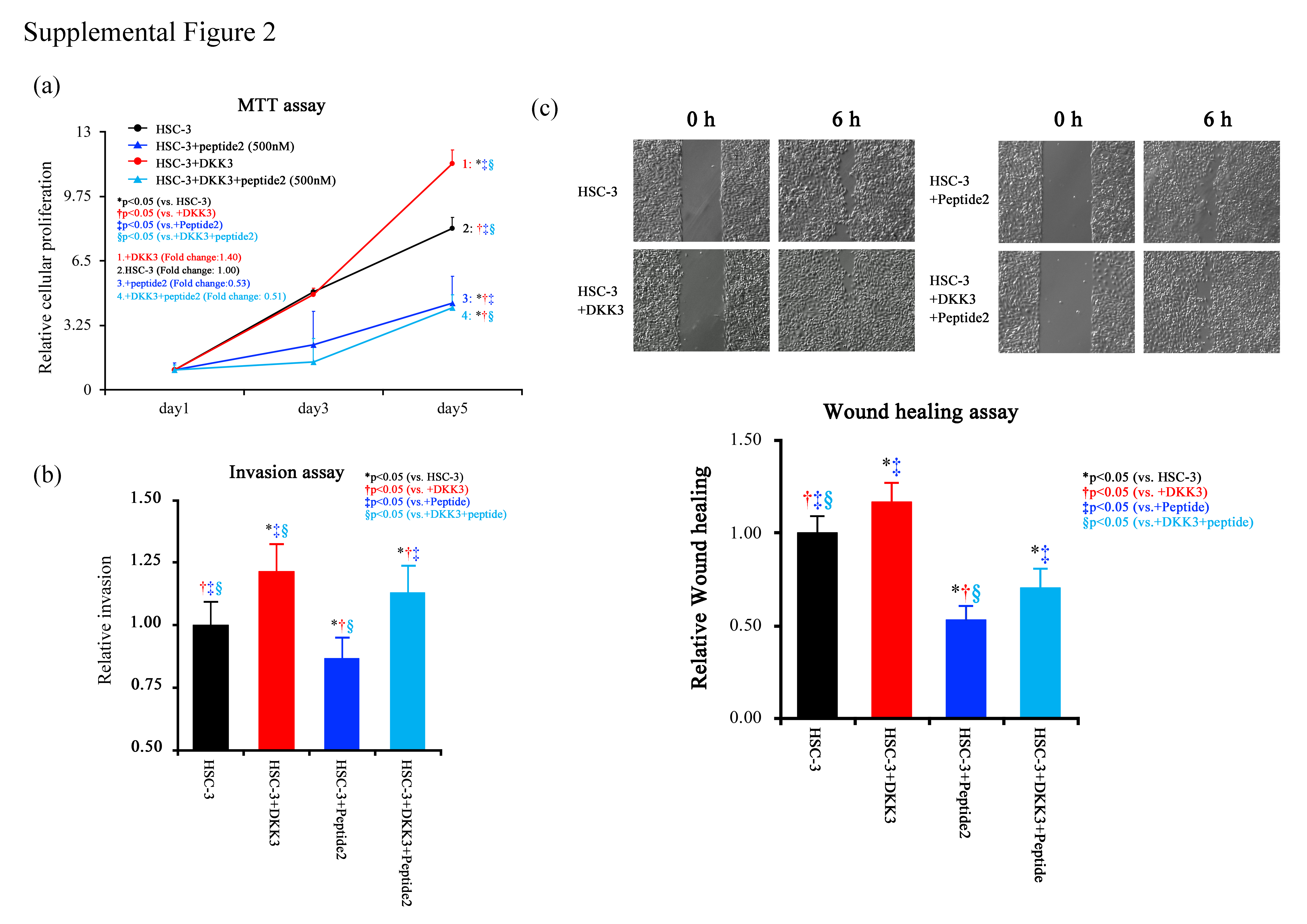

Supplement: Supplementary file 2 — Additional file 2: Figure S2. The effects of peptide2 on cellular proliferation, invasion, and migration. a–c DKK3 over-expression significantly elevated cellular proliferation, invasion, and migration. Administration of peptide2 (500 nM) significantly suppressed cellular proliferation, invasion, and migration and canceled the elevating effects of DKK3 transfection on them. [file 12935_2022_2783_MOESM2_ESM.jpg]

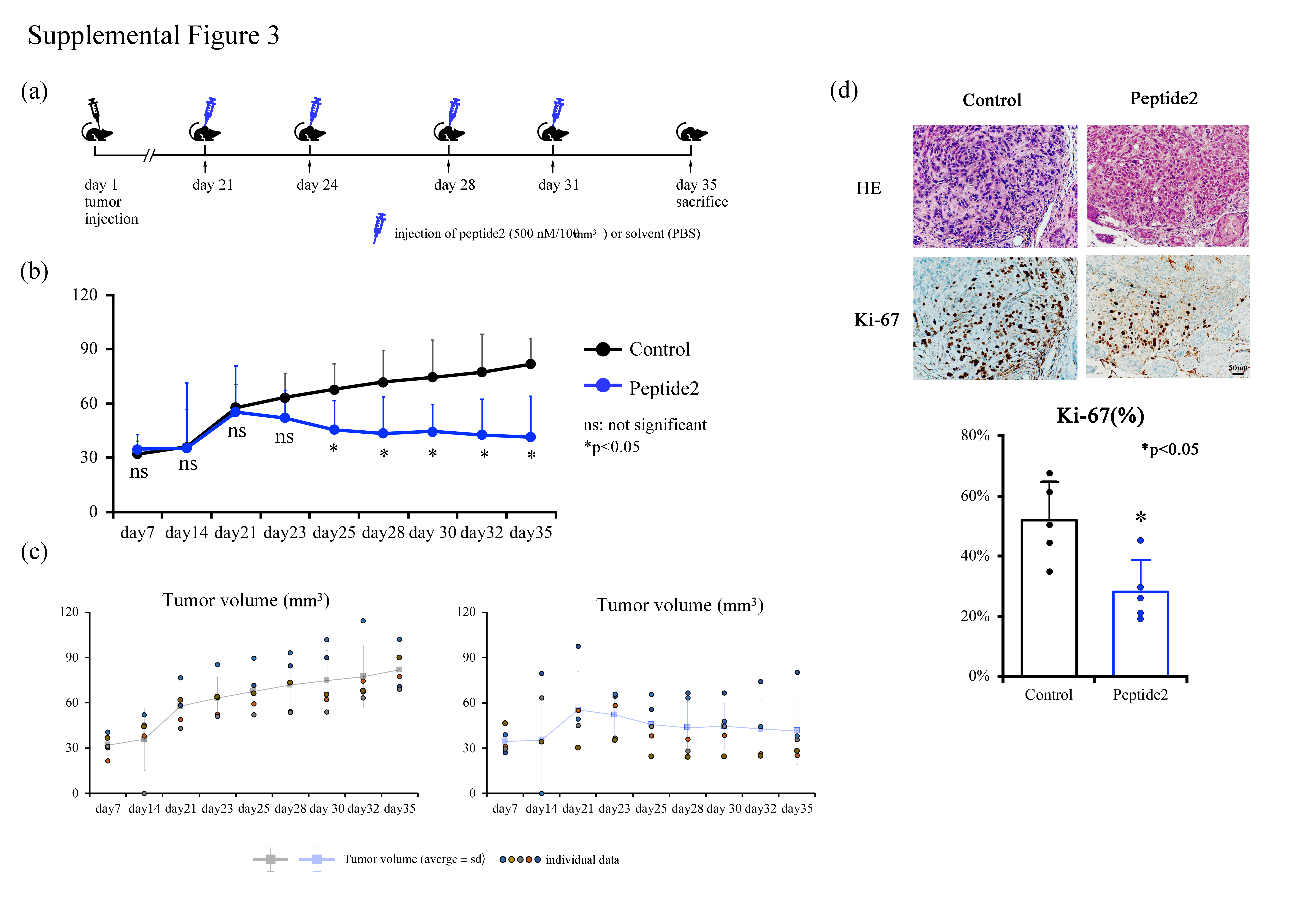

Supplement: Supplementary file 3 — Additional file 3: Figure S3. The therapeutic effect of peptide2 in the xenograft model. (a) Schematic explanation for the schedule of the animal experiments. (b) The tumor volume was significantly reduced by the administration of peptide from days 25 to 35. (c) The tumor volume of the individual mice is shown. The average ± standard deviation (sd) is also displayed. (d) There were no histological differences between the control and peptide groups, but the Ki-67 index of the peptide group was significantly decreased compared to the control group. [file 12935_2022_2783_MOESM3_ESM.jpg]
